# Symptom-Specific Factors Associated With Chronic Constipation in Older Adults: Cross-Sectional Survey of Patient-Reported Outcomes

**DOI:** 10.2196/93531

**Published:** 2026-07-10

**Authors:** Michinobu Takenaka, Toshinari Mitsuoka, Hidehiko Sakurai

**Affiliations:** 1 Shirakaba Pharmacy Co. Ltd, Hokkaido University of Science, Sapporo, Hokkaido, Japan; 2 Faculty of Pharmaceutical Sciences, Hokkaido University of Science, 15-4-1, Maeda 7-jo, Teine-ku, Sapporo, Hokkaido, 006-8585, Japan, 81 11-676-8728, 81 11-676-8666

**Keywords:** constipation, older adults, laxative therapy, patient-reported outcomes, gastroenterology, bowel, digestive

## Abstract

**Background:**

In older adults with chronic constipation, symptoms of defecation difficulty often persist despite improvements in bowel movement frequency. However, constipation-related symptoms have rarely been examined as independent outcomes.

**Objective:**

The objective of this study is to explore factors associated with constipation-related symptoms, less than 3 spontaneous bowel movements per week, straining during bowel movements, a sensation of incomplete evacuation, and a sensation of anorectal obstruction or blockage.

**Methods:**

A cross-sectional web-based survey was conducted to collect patient-reported data. Constipation-related symptoms were defined according to the Rome IV criteria. Each symptom was treated as a binary variable in the analyses. Multivariable logistic regression analyses were used to examine associations between stool characteristics and medication, treatment processes, and lifestyle-related factors. Stool characteristics were classified using the Bristol Stool Form Scale, with hard stools defined as types 1 and 2.

**Results:**

We enrolled 546 participants and investigated the following constipation symptoms: less than 3 spontaneous bowel movements per week (n=226, 41.4%), straining (n=330, 60.4%), sensation of incomplete evacuation (n=344, 63.0%), and sensation of anorectal obstruction or blockage (n=242, 44.3%). Multivariable logistic regression analyses for these outcome variables showed that hard stool was consistently and independently associated with all constipation-related symptoms (odds ratio [OR] 1.86‐4.66). Symptom-specific associations that remained significant after false discovery rate adjustment included lower physical activity with less than 3 spontaneous bowel movements per week (OR 0.86, 95% CI 0.77‐0.96), stimulant laxative use with the sensation of incomplete evacuation (OR 2.01, 95% CI 1.22‐3.31), and lower adherence to the prescribed dosage with the sensation of anorectal obstruction or blockage (OR 0.87, 95% CI 0.78‐0.97). In contrast, the experience of adverse effects was associated with a higher probability of less than 3 spontaneous bowel movements per week and the sensation of anorectal obstruction or blockage.

**Conclusions:**

In older adults with chronic constipation, different constipation-related symptoms showed distinct patterns of association, while hard stool consistency emerged as a common factor across symptoms. These exploratory findings may support symptom-oriented assessment and help inform individualized management approaches using electronically collected patient-reported outcomes.

## Introduction

Chronic constipation is a functional gastrointestinal disorder frequently observed in older adults. It is a heterogeneous syndrome characterized by multiple symptoms, including reduced bowel movement frequency, straining during defecation, a sensation of incomplete evacuation, and anal obstruction [1]. In older adults, a combination of age-related decreases in intestinal motility, reduced physical activity, and polypharmacy contributes to the complexity of symptom presentation and treatment responses [2].

Consequently, in older adults with chronic constipation, symptoms such as straining during defecation, the sensation of incomplete evacuation, and the sensation of anorectal obstruction or blockage may persist even when the frequency of bowel movements is sufficient [3,4]. These symptoms are included in the Rome IV diagnostic criteria for chronic constipation [5]. However, many prior studies have primarily evaluated the frequency of spontaneous bowel movements [6] and the severity of constipation [7] as primary outcomes. Although individualized management tailored to specific symptoms is increasingly important, evidence regarding factors associated with individual symptoms remains limited [8].

In addition, stool consistency, particularly hard stools, has been shown to be a pathophysiological factor potentially involved in reduced stool frequency and in common defecation disorder symptoms, such as straining, the sensation of incomplete evacuation, and anal obstruction [9].

Meanwhile, treatment process elements (eg, experience of adverse effects, adherence to prescribed dosages, and shared treatment goals) and lifestyle factors (eg, fluid intake and physical activity) have been suggested to be associated with symptoms, in addition to pharmacotherapy, in the management of chronic constipation [10]. However, few studies have systematically integrated clinical characteristics, treatment processes, and lifestyle habits into a single analytical model to examine their symptom-specific associations within the same cohort.

Therefore, this study employed a cross-sectional design targeting adults aged 65 to 74 years with chronic constipation who were receiving treatment. We collected patient-reported data from a web-based survey and aimed to explore the relationship between individual constipation-related symptoms and clinically relevant factors under pharmacological treatment. This study was not designed to test any specific pre-existing hypotheses but was rather planned as an exploratory study to generate clinical hypotheses for future investigation by identifying factors associated with various symptoms in patients with chronic constipation. This approach may help characterize each symptom, thereby providing insights that could contribute to the development of more individualized management strategies through digital tools, beyond focusing solely on bowel movement frequency.

## Methods

### Study Design and Participants

This cross-sectional study was based on an anonymous, self-administered, web-based questionnaire survey conducted in Japan in July 2024. After obtaining approval from the Institutional Review Board, participants were recruited through Macromill, Inc, a research panel company that maintains a nationwide panel of registered monitors, including a disease-specific panel of patients with chronic constipation. This study was conducted as a closed web-based survey, and members of the chronic constipation panel were invited through email using a consecutive convenience sampling method. The purpose and content of the study were explained on the survey screen, and only participants who provided consent were included in the survey. The participants were older adults aged 65 to 74 years who took constipation medications prescribed by medical institutions for at least 4 days per week, excluding those who used the medication on an as-needed basis. The reason for limiting the target age range from 65 to 74 years was to ensure the reliability of self-reported data in an online survey and to minimize the influence of severe cognitive decline and marked physical functional impairment, which are more common among adults aged 75 years and older.

Participants with restricted fluid intake, diet, or physical activity due to physician instructions were excluded because these medically prescribed restrictions might prevent actual behaviors from accurately reflecting usual lifestyle habits, thereby potentially confounding the assessment of lifestyle factors.

### Measurements

The constipation-related symptoms, stool consistency, treatment process factors, and lifestyle factors analyzed in this study are listed in Table 1. In this study, each variable was operationally defined based on the respondent’s self-reported assessment of the corresponding questionnaire item. Details of the questionnaire items are provided in Table S1 in Multimedia Appendix 1.

**Table 1.** Variables and their definitions used in the study.

Category and variables	Definition or categorization
Outcome variables	
Fewer than 3 spontaneous bowel movements per week	Presence of less than 3 spontaneous bowel movements per week (yes/no)
Straining during bowel movements	Presence of straining in ≥1 out of 4 bowel movements (yes/no)
Sensation of incomplete evacuation	Presence of sensation of incomplete evacuation (yes/no)
Sensation of anorectal obstruction or blockage	Presence of sensation of anorectal obstruction or blockage (yes/no)
Manual maneuvers to facilitate defecation	Presence of manual maneuvers to facilitate defecation (yes/no)
Demographic factor	
Age (y)	65 to 74 years (continuous)
Sex	Male/female
Stool-related factor	
Hard stool	BSFS^a^ type 1‐2 versus others
Medical factors	
Specialty of prescribing physician	Gastroenterology/coloproctology versus others
Experience of adverse effects	Yes/no
Medication use	
Osmotic laxatives	Yes/no
Stimulant laxatives	Yes/no
Gastric acid–suppressing agents	Yes/no
Treatment process factors	
Shared treatment goals	7-point scale (1=“strongly disagree” to 7=“strongly agree”)
Adherence to prescribed dosage	7-point scale (1=“never” to 7=“always”)
Lifestyle factors	
Probiotic intake	7-point scale (1=“absolutely not applicable” to 7=“absolutely applicable”)
Physical activity	
Dietary fiber intake	
Daily fluid intake	

a BSFS: Bristol Stool Form Scale.

#### Outcome Variables

The following outcome variables were defined: less than 3 spontaneous bowel movements per week, straining during bowel movements, sensation of incomplete evacuation, sensation of anorectal obstruction or blockage, and the presence or absence of manual maneuvers to facilitate defecation (defined as one of the symptoms in the Rome IV criteria). Each constipation-related symptom was dichotomized as 1 (“present”) or 0 (“absent”). These symptoms were defined based on the symptom definitions of chronic constipation according to the Rome IV criteria [11]. However, outcomes with a low number of events were excluded from the multivariable analysis because they might not meet the event count required for multivariable logistic regression analysis.

#### Explanatory Variables

Sex was included as a basic attribute in this analysis. Sex was treated as a covariate because previous studies have reported that it can influence the pathophysiology and symptom profile of individuals with chronic constipation [1].

Stool consistency was assessed using the Bristol Stool Form Scale (BSFS) and classified as hard stool (types 1‐2) or otherwise [12]. Hard stools are a pathological factor that may cause multiple constipation symptoms. Therefore, in this study, they were included as an explanatory background factor, independent of symptom outcome variables, and were used to examine the factors associated with specific symptoms.

Medical-related factors were classified based on the prescribing department as gastroenterology, digestive medicine, proctology, or others. Additionally, adverse effects associated with constipation medications were operationally defined as self-reported experiences of symptoms (eg, severe diarrhea or abdominal pain) attributed to laxative use. Participants were asked whether they had experienced any such side effects using a yes/no response format. Responses were dichotomized as “present” when participants reported experiencing any of these symptoms, and “absent” otherwise. No further subtype definitions or severity grading criteria were applied to minimize respondent burden in this online survey of older adults. Medication usage status, specifically the use of osmotic laxatives (saline laxatives), stimulant laxatives, and gastric acid secretion inhibitors, was also assessed. The type of constipation medication used was classified based on multiple-choice and open-ended questions. Acid secretion inhibitors were included as background factors in the analysis because they have been suggested to affect the intestinal microbiota and stool characteristics, potentially correlating with constipation symptoms and defecation abnormalities [13-15].

The treatment process factors included shared treatment goals and adherence to the prescribed dosages. Specifically, these items were developed with reference to the Necessity-Concerns Framework [8], an academic framework describing treatment behaviors and medication beliefs in patients with chronic conditions. These factors are treatment process elements potentially related to patients’ understanding of treatment and medication awareness and have been suggested to influence medication behavior and treatment continuity in long-term therapy [16].

Shared treatment goals were assessed using the question, “Do you feel you share treatment goals for constipation symptoms with your prescribing physician?” Responses were recorded on a 7-point scale ranging from 1 (“strongly disagree”) to 7 (“strongly agree”).

Adherence to the prescribed dosage was assessed using the question, “How often do you follow the prescribed dosage and frequency of your constipation medications or laxatives as instructed by your physician or pharmacist?” Responses were recorded on a 7-point scale ranging from 1 (“never”) to 7 (“always”).

Lifestyle factors, including the consumption of lactic-acid bacterial products, exercise habits, dietary fiber intake, and daily fluid intake, were selected based on recommendations from international organizations (American Gastroenterological Association–American College of Gastroenterology and World Gastroenterology Organization) and Japanese clinical practice guidelines for chronic constipation [17-19], as well as reviews on nonpharmacological interventions for managing chronic constipation [20-23]. The detailed operational definitions and questionnaire items used to assess lifestyle factors are shown in Table S1 in Multimedia Appendix 1. Specifically, each factor was operationalized as the participant’s self-reported degree of implementation of the corresponding constipation-related lifestyle behavior in daily life. Each item was assessed using a 7-point scale ranging from 1 (“absolutely not applicable”) to 7 (“absolutely applicable”).

Questionnaire items related to lifestyle factors, shared treatment goals, and adherence were subjected to a content validity review by an interdisciplinary expert panel consisting of 6 members (3 pharmacists, 1 general practitioner, 1 regulatory science specialist, and 1 medication adherence researcher). The panel evaluated the clarity, clinical relevance, and appropriateness of each item.

Given that the target population comprised adults aged 65 to 74 years and that the survey was administered online, a concise questionnaire design was adopted to minimize respondent burden and maintain response quality. Previous methodological research has suggested that single-item measures may be appropriate when the objective is to capture respondents’ overall subjective appraisal of clearly interpretable constructs in large-scale surveys [24]. As this study focused on global perceptions rather than detailed multidimensional assessments of each construct, each variable was evaluated using a single-item scale.

### Statistical Analysis

Descriptive statistics are presented as mean (SD) for continuous variables and as numbers and percentages for categorical variables. Multivariable logistic regression analyses were performed for each constipation-related symptom. All variables were entered simultaneously into the model using the forced-entry method. This approach was used to estimate the independent association of each factor with each symptom after adjustment for the other covariates. In the multivariable analysis, odds ratios (ORs) and 95% CIs were calculated. The overall performance of the multivariable logistic regression models was evaluated using the area under the receiver operating characteristic curve (AUC) and the Hosmer-Lemeshow goodness-of-fit test. To account for the increased risk of type I error associated with multiple exploratory outcome analyses, a multiplicity adjustment was applied. *P* values were corrected using the Benjamini-Hochberg method to control the false discovery rate (FDR). Given that this study is exploratory in nature, this approach was determined to be more appropriate than a stricter family-wise error correction, as it helps suppress false-positive findings while reducing the risk of overlooking potentially relevant factors. Statistical significance was defined as an adjusted *P* value of less than .05. IBM SPSS Statistics version 30.0 and R version 4.6 (R Foundation for Statistical Computing) were used for statistical analysis. However, outcomes with a low number of events were excluded from the multivariable analysis because they might not meet the required event count for multivariable logistic regression analysis.

As a supplementary analysis, we evaluated the associations between symptoms and each explanatory variable using univariate analysis. The chi-square test was used for categorical variables, and the two-tailed *t* test was used for continuous variables assuming a normal distribution. All analyses were conducted using the same cohort of participants (N=546), and separate multiple regression models were constructed for each constipation-related symptom. No a priori sample size calculation was performed, and all available data were included. A post hoc assessment using G*Power version 3.1.9.7 (Heinrich-Heine-Universität Düsseldorf) indicated that the sample size was sufficient for the planned analyses.

### Ethical Considerations

This study was conducted in compliance with the World Medical Association Declaration of Helsinki and was approved by the Ethics Committee of Hokkaido University of Science (approval number 24‐06). All participants voluntarily and anonymously completed the survey and were informed in advance that the submission of their responses would be considered consent to participate. Participants received written explanations prior to participation, and consent was confirmed by submitting responses to the online survey. All data were collected anonymously through an online survey, and no personally identifiable information was obtained. Appropriate measures were taken to ensure data privacy and confidentiality throughout the study. Participants received compensation through the survey panel provider in accordance with its standard incentive policy.

## Results

### Participants

Of the 824 panel members invited to participate in the survey, 554 responded. After excluding 8 cases in which the type of laxative could not be identified, 546 subjects were analyzed. The mean age of participants was 69.4 (SD 2.9) years, and 66.8% (365/546) were men. The prescribing department for laxatives was gastroenterology/coloproctology in 24.5% (134/546) of cases. Side effects of laxatives were experienced by 22.2% (121/546) of patients, and hard stools were reported by 23.1% (126/546) of patients. Among the participants, 41.4% (226/546) had less than 3 spontaneous bowel movements per week, 60.4% (330/546) experienced straining during bowel movements, 63.0% (344/546) experienced a sensation of incomplete evacuation, and 44.3% (242/546) reported a sensation of anorectal obstruction or blockage. In total, 6.4% (35/546) of patients required maneuvers to facilitate their bowel movements. Detailed background information on the participants is provided in Table 2.

**Table 2.** Patient characteristics (N=546).

Characteristics	Participants
Outcome variables, n (%)	
Frequency of spontaneous bowel movements	226 (41.4)
Straining during bowel movements	330 (60.4)
Sensation of incomplete evacuation	344 (63.0)
Sensation of anorectal obstruction or blockage	242 (44.3)
Manual maneuvers to facilitate defecation	35 (6.4)
Demographic factors	
Age (y), mean (SD)	69.4 (2.9)
Male sex, n (%)	365 (66.8)
Female sex, n (%)	181 (33.2)
Stool factor	
Hard stool (BSFS^a^ type 1‐2), n (%)	126 (23.1)
Medical factors, n (%)	
Gastroenterology/coloproctology	134 (24.5)
Experience of adverse effects from constipation medications	121 (22.2)
Medication use, n (%)	
Osmotic laxatives (a salt laxative)	390 (71.4)
Stimulant laxatives	166 (30.4)
Gastric acid–suppressing agents	168 (30.8)
Treatment process factors (Likert scale, 1‐7), mean (SD)	
Shared treatment goals	5.37 (1.25)
Adherence to prescribed dosage	5.94 (1.67)
Lifestyle factors (Likert scale, 1‐7), mean (SD)	
Probiotic intake	4.75 (1.77)
Physical activity	3.94 (1.77)
Dietary fiber intake	4.71 (1.20)
Fluid intake	4.39 (1.51)

a BSFS: Bristol Stool Form Scale.

### Univariate Analysis

In the univariate analysis, the group with less than 3 spontaneous bowel movements per week showed significantly higher proportions of women, those with adverse effects and hard stools, as well as significantly lower levels of physical activity, dietary fiber intake, and daily fluid intake. Straining during bowel movements was significantly more common in the hard-stool group and was significantly associated with lower levels of shared treatment goals and daily fluid intake. The sensation of incomplete evacuation was more common among those who experienced adverse effects in the hard-stool group and among stimulant laxative users. The sensation of anorectal obstruction or blockage was significantly more frequent in the hard-stool group, among those who experienced adverse effects, and in the group with low adherence to the prescribed dose (Tables S2A-S2E in Multimedia Appendix 1).

### Multivariable Logistic Regression Analysis

Multivariable analysis revealed that each constipation-related symptom had a distinct set of factors that remained significantly associated after adjustment for other variables (Tables3^6^).

**Table 3.** Binary logistic regression analysis of factors associated with having less than 3 spontaneous bowel movements per week^a^.

Explanatory variables	OR^b^ (95% CI)	*P* value (adjusted for covariates)	*q* value^c^ (FDR^d^-adjusted)
Demographic factor			
Sex (female vs male [reference])	1.88 (1.27‐2.78)	.002	.004
Stool-related factor			
Hard stool (BSFS^e^ type 1‐2)	2.15 (1.40‐3.30)	<.001	.001
Medical factor			
Specialty of prescribing physician (gastroenterology or coloproctology vs others [reference])	1.14 (0.75‐1.75)	.53	.83
Experience of adverse effects from constipation medications (yes vs no [reference])	2.00 (1.30‐3.07)	.002	.008
Medication use			
Osmotic laxatives (yes vs no [reference])	0.71 (0.44‐1.14)	.15	.28
Stimulant laxatives (yes vs no [reference])	0.99 (0.62‐1.59)	.96	.96
Gastric acid–suppressing agents (yes vs no [reference])	1.07 (0.72‐1.59)	.74	.74
Treatment process factor			
Shared treatment goals	0.96 (0.83‐1.11)	.57	.57
Adherence to prescribed dosage	0.96 (0.86‐1.08)	.51	.58
Lifestyle factor			
Probiotic intake	1.00 (0.90‐1.12)	.99	.99
Physical activity	0.86 (0.77‐0.96)	.007	.04
Dietary fiber intake	0.93 (0.79‐1.11)	.43	.86
Daily fluid intake	0.92 (0.81‐1.04)	.20	.27

a All explanatory variables were entered simultaneously into the multivariable logistic regression model, regardless of univariable significance. Area under the receiver operating characteristic curve: 0.685 (95% CI 0.640‐0.731). Hosmer-Lemeshow test: *P*=.38.

b OR: odds ratio.

c *q* values were calculated using the Benjamini-Hochberg method to control the false discovery rate (FDR) at <.05 across 4 independent models.

d FDR: false discovery rate.

e BSFS: Bristol Stool Form Scale.

**Table 4.** Binary logistic regression analysis of factors associated with straining during bowel movements^a^.

Explanatory variables	OR^b^ (95% CI)	*P* value (adjusted for covariates)	*q* value^c^ (FDR^d^-adjusted)
Demographic factor			
Sex (female vs male [reference])	0.52 (0.34‐0.77)	.001	.004
Stool-related factor			
Hard stool (BSFS^e^ type 1‐2)	4.66 (2.74‐7.92)	<.001	.001
Medical factor			
Specialty of prescribing physician (gastroenterology or coloproctology vs others [reference])	1.21 (0.78‐1.88)	.39	.83
Experience of adverse effects from constipation medications (yes vs no [reference])	1.49 (0.94‐2.35)	.09	.09
Medication use			
Osmotic laxatives (yes vs no [reference])	1.24 (0.75‐2.03)	.40	.40
Stimulant laxatives (yes vs no [reference])	1.49 (0.91‐2.45)	.11	.15
Gastric acid–suppressing agents (yes vs no [reference])	0.92 (0.62‐1.38)	.69	.74
Treatment process factor			
Shared treatment goals	0.87 (0.74‐1.02)	.09	.18
Adherence to prescribed dosage	0.95 (0.84‐1.06)	.34	.68
Lifestyle factor			
Probiotic intake	1.04 (0.94‐1.17)	.45	.99
Physical activity	0.92 (0.82‐1.03)	.15	.30
Dietary fiber intake	1.04 (0.87‐1.24)	.68	.88
Daily fluid intake	0.88 (0.77‐1.00)	.047	.19

a All explanatory variables were entered simultaneously into the multivariable logistic regression model, regardless of univariable significance. Area under the receiver operating characteristic curve 0.708 (95% CI 0.664‐0.752). Hosmer-Lemeshow test: *P*=.02.

b OR: odds ratio.

c *q* values were calculated using the Benjamini-Hochberg method to control the false discovery rate (FDR) at <.05 across 4 independent models.

d FDR: false discovery rate.

e BSFS: Bristol Stool Form Scale.

**Table 5.** Binary logistic regression analysis of factors associated with the sensation of incomplete evacuation^a^.

Explanatory variables	OR^b^ (95% CI)	*P* value (adjusted for covariates)	*q* value^c^ (FDR^d^ -adjusted)
Demographic factor			
Sex (female vs male [reference])	0.98 (0.66‐1.45)	.91	.91
Stool-related factor			
Hard stool (BSFS^e^ type 1‐2)	1.86 (1.18‐2.92)	.008	.008
Medical factor			
Specialty of prescribing physician (gastroenterology or coloproctology vs others [reference])	1.08 (0.71‐1.65)	.72	.83
Experience of adverse effects from constipation medications (yes vs no [reference])	1.56 (0.99‐2.46)	.05	.07
Medication use			
Osmotic laxatives (yes vs no [reference])	1.36 (0.84‐2.22)	.21	.28
Stimulant laxatives (yes vs no [reference])	2.01 (1.22‐3.31)	.006	.02
Gastric acid–suppressing agents (yes vs no [reference])	1.22 (0.82‐1.82)	.32	.74
Treatment process factor			
Shared treatment goals	0.85 (0.73‐0.99)	.04	.16
Adherence to prescribed dosage	0.98 (0.88‐1.10)	.78	.78
Lifestyle factor			
Probiotic intake	1.01 (0.91‐1.13)	.79	.99
Physical activity	0.99 (0.88‐1.11)	.84	.84
Dietary fiber intake	0.99 (0.83‐1.17)	.88	.88
Daily fluid intake	0.90 (0.80‐1.03)	.12	.24

a All explanatory variables were entered simultaneously into the multivariable logistic regression model, regardless of univariable significance. Area under the receiver operating characteristic curve 0.647 (95% CI 0.600‐0.695). Hosmer-Lemeshow test: *P*=.85.

b OR: odds ratio.

c *q* values were calculated using the Benjamini-Hochberg method to control the false discovery rate (FDR) at <.05 across 4 independent models.

d FDR: false discovery rate.

e BSFS: Bristol Stool Form Scale.

**Table 6.** Binary logistic regression analysis of factors associated with the sensation of anorectal obstruction or blockage^a^.

Explanatory variables	OR^b^ (95% CI)	*P* value (adjusted for covariates)	*q* value^c^ (FDR^d^-adjusted)
Demographic factor			
Sex (female vs male [reference])	0.66 (0.44‐0.98)	.04	.053
Stool-related factor			
Hard stool (BSFS^e^ type 1‐2)	2.73 (1.78‐4.20)	<.001	.001
Medical factor			
Specialty of prescribing physician (gastroenterology or coloproctology vs others [reference])	0.96 (0.63‐1.46)	.83	.83
Experience of adverse effects from constipation medications (yes vs no [reference])	1.69 (1.10‐2.59)	.02	.04
Medication use			
Osmotic laxatives (yes vs no [reference])	1.59 (0.98‐2.57)	.06	.24
Stimulant laxatives (yes vs no [reference])	1.52 (0.94‐2.46)	.09	.15
Gastric acid–suppressing agents (yes vs no [reference])	1.19 (0.80‐1.76)	.39	.74
Treatment process factor			
Shared treatment goals	0.91 (0.78‐1.05)	.19	.25
Adherence to prescribed dosage	0.87 (0.78‐0.97)	.01	.04
Lifestyle factor			
Probiotic intake	1.02 (0.92‐1.14)	.70	.99
Physical activity	0.96 (0.86‐1.07)	.42	.56
Dietary fiber intake	0.89 (0.75‐1.06)	.19	.76
Daily fluid intake	1.01 (0.89‐1.15)	.87	.87

a All explanatory variables were entered simultaneously into the multivariable logistic regression model, regardless of univariable significance. Area under the receiver operating characteristic curve 0.682 (95% CI 0.637‐0.727). Hosmer-Lemeshow test: *P*=.59.

b OR: odds ratio.

c *q* values were calculated using the Benjamini-Hochberg method to control the false discovery rate (FDR) at <.05 across 4 independent models.

d FDR: false discovery rate.

e BSFS: Bristol Stool Form Scale.

Having less than 3 spontaneous bowel movements per week was significantly associated with hard stool (BSFS type 1‐2; OR 2.15, 95% CI 1.40‐3.30) and the experience of adverse effects from constipation medications (OR 2.00, 95% CI 1.30‐3.07). Furthermore, women were significantly associated with the outcome, with higher odds than men (OR 1.88, 95% CI 1.27‐2.78). In contrast, higher levels of physical activity were significantly associated with a lower likelihood of having less than 3 spontaneous bowel movements per week (OR 0.86, 95% CI 0.77‐0.96) and were identified as an independent factor associated with less than 3 spontaneous bowel movements per week.

Straining during bowel movements was most strongly associated with hard stool (OR 4.66, 95% CI 2.74‐7.92), whereas female sex was associated with lower odds of straining (OR 0.52, 95% CI 0.34‐0.77). Higher daily fluid intake was also associated with lower odds of straining during bowel movements in the multivariable-adjusted model (OR 0.88, 95% CI 0.77‐1.00); however, this association did not remain significant after FDR adjustment (*q*=.19). The experience of adverse effects from constipation medications was not statistically significantly associated with straining during bowel movements, although the point estimate suggested a possible positive association (OR 1.49, 95% CI 0.94‐2.35; *q*=.09).

Regarding the sensation of incomplete evacuation, hard stool (OR 1.86, 95% CI 1.18‐2.92) and stimulant laxative use (OR 2.01, 95% CI 1.22‐3.31) were significantly associated with higher odds of incomplete evacuation. In contrast, greater shared treatment goals were associated with lower odds (OR 0.85, 95% CI 0.73‐0.99), although this association did not remain significant after FDR adjustment (*q*=.16). The experience of adverse effects was not statistically significantly associated with the sensation of incomplete evacuation, although the point estimate suggested a possible positive association (OR 1.56, 95% CI 0.99‐2.46; *q*=.07).

Finally, regarding the sensation of anorectal obstruction or blockage, hard stool (OR 2.73, 95% CI 1.78‐4.20) and the experience of adverse effects from laxatives (OR 1.69, 95% CI 1.10‐2.59) were significantly associated factors. In contrast, adherence to the prescribed dosage was significantly and inversely associated with the sensation of anorectal obstruction or blockage (OR 0.87, 95% CI 0.78‐0.97). While female sex was associated with lower odds than male sex (OR 0.66, 95% CI 0.44‐0.98), this association, however, did not reach statistical significance after FDR adjustment (*q*=.053).

Manual maneuvers to facilitate defecation were excluded from the multivariable analysis because of the low number of events.

All models showed moderate discriminative ability, with AUC values ranging from 0.647 to 0.708. The Hosmer-Lemeshow test indicated no significant lack of fit for the models of low defecation frequency, sensation of incomplete evacuation, and anorectal obstruction. In contrast, the straining model showed a significant result (*P*=.02), with an AUC of 0.708. In a sensitivity analysis using reversed outcome coding, the Hosmer-Lemeshow test for the straining model became nonsignificant (*P*=.17).

## Discussion

### Principal Findings

This study examined factors associated with individual chronic constipation-related symptoms in adults aged 65 to 74 years with chronic constipation who were receiving pharmacological treatment, using a cross-sectional web-based survey. The findings suggest that these symptoms are not explained by a single underlying factor. Hard stool emerged as a primary factor associated with multiple symptoms, whereas treatment process elements and lifestyle factors contributed to symptom-specific variations. Overall, the clinical interpretation of chronic constipation in this population should take into account both shared and symptom-specific associated factors, rather than relying solely on isolated statistical associations.

### Significance of Hard Stool as a Common Factor

Although hard stools are clinically classified as constipation-related symptoms, they were treated in this study as a stool-related characteristic reflecting defecation status. Hard stool (BSFS type 1‐2) was associated with the frequency of spontaneous bowel movements and subjective symptoms, such as straining during bowel movements, the sensation of incomplete evacuation, and the sensation of anorectal obstruction or blockage.

Notably, the strength of these associations differed across symptoms. Hard stool was more strongly associated with straining during bowel movements than with the sensation of incomplete evacuation. This difference may reflect underlying pathophysiological mechanisms. Straining is primarily related to increased resistance during stool expulsion and is therefore highly dependent on stool consistency [25,26]. Hard stool, reflecting prolonged intestinal transit time [2], may increase mechanical resistance during defecation, thereby resulting in a stronger association with straining. In contrast, the sensation of incomplete evacuation is not solely determined by stool characteristics but is also related to rectal sensory function and symptom perception. Previous studies have suggested that this sensation does not always correspond to objective physiological measures, such as colonic transit time or anorectal function [25-30], which may explain the relatively weaker association observed in this study.

In older adults, age-related factors such as reduced food intake and polypharmacy have been reported to contribute to stool hardening by prolonging intestinal transit time and altering intestinal motility [2]. Hard stool formation may contribute to difficulty in defecation or fecal impaction in the rectum [5,11]. These findings support the use of standardized measures, such as the BSFS, to evaluate stool consistency as a clinically relevant therapeutic target.

### Experience of Adverse Effects as a Common Factor

Here, adverse effects were associated with the frequency of spontaneous bowel movements and the sensation of anorectal obstruction or blockage. These associations may be clinically meaningful because the frequency of spontaneous bowel movements is susceptible to the effects of drug therapy and is known to fluctuate rapidly due to the discontinuation of medication or self-adjustment [31]. Additionally, the sensation of anorectal obstruction or blockage reflects instability during evacuation. If the effects of drug therapy are inconsistent, symptoms may persist [25,27]. Conversely, the absence of clear associations between adverse effects and straining during bowel movements or the sensation of incomplete evacuation may suggest that these symptoms are influenced more strongly by defecatory mechanisms. These symptoms are primarily attributable to defecatory disorders, such as pelvic floor dysfunction [25,27], and are therefore more likely to be influenced by the defecatory mechanism itself rather than by the consistency of drug efficacy. This may reflect the inclusion of patients for whom pharmacological treatment alone was insufficient. The relationship between symptoms and adverse effects may be confounded by other treatment process factors, such as daily fluid intake and shared treatment goals, which may independently influence symptoms such as straining and incomplete evacuation.

### Fewer Than 3 Spontaneous Bowel Movements Per Week and Physical Activity

Among the lifestyle factors examined, physical activity emerged as a factor associated with spontaneous bowel movement frequency. Constipation characterized by reduced bowel movement frequency primarily involves prolonged colonic transit time and decreased intestinal motility [11,25,28]. Previous studies targeting physically inactive middle-aged patients with chronic constipation have suggested that exercise interventions are associated with intestinal motility and colonic transit time. However, consistent results regarding bowel movement frequency have not yet been obtained [32].

The observed association may indicate the clinical relevance of physical activity in relation to bowel movement frequency in older adults. Future research should clarify the role of nonpharmacological therapies through evaluations that consider both physical activity levels and functional capacity.

### Straining During Bowel Movements and Daily Fluid Intake

Daily fluid intake was associated with reduced straining during defecation before FDR correction; however, this association was not significant after adjustment for multiple comparisons. Straining during defecation results from increased resistance to stool passage and is known to be strongly influenced by stool consistency [25,26]. Previous studies have reported that insufficient fluid intake contributes to stool hardening [29]. The findings suggest a possible association between daily fluid intake and straining during defecation through its influence on stool consistency. However, this interpretation warrants caution because the association did not remain significant after adjustment for multiple comparisons.

Although the statistical evidence was limited, the assessment of daily fluid intake may still be clinically useful when evaluating straining during defecation.

### Sensation of Incomplete Evacuation and Shared Treatment Goals

Although the observed association should be interpreted cautiously because it did not remain significant after correction for multiple testing, it may still provide insight into how patients perceive constipation-related symptoms. Studies involving patients with severe chronic constipation have reported that subjective symptoms, such as a sense of incomplete evacuation, do not necessarily correspond to objective physiological markers, including colonic transit time and anorectal function [28,30]. This discrepancy suggests that subjective symptom perception may be influenced by physiological dysfunction and by patients’ expectations and their understanding of the treatment. Therefore, while the associations observed in this study are exploratory, sharing treatment goals may be associated with how patients perceive a sense of incomplete evacuation. When evaluating and managing a sense of incomplete evacuation, an assessment framework that incorporates both patient-reported perceptions—including their understanding of treatment goals—and underlying pathophysiology may be clinically useful.

### Sensation of Anorectal Obstruction or Blockage and Adherence to Prescribed Dosage

The observed association between the sensation of anorectal obstruction or blockage and adherence to the prescribed dosage suggests that the stability of drug efficacy may play a role in modulating defecatory symptoms. Anorectal obstruction or blockage is reported to be related to the instability of anorectal function and bowel movements [11,28]. Adherence to the prescribed dosage may be associated with the stability of drug efficacy, thereby potentially modulating symptoms. However, the experience of adverse effects frequently results in medication discontinuation, which may subsequently disrupt bowel habits and influence symptoms. The observed relationship between adherence to the prescribed regimen and symptoms suggests that consistent medication use may help regulate the defecation process and may therefore be associated with a reduced sensation of anorectal obstruction or blockage. Accordingly, a comprehensive evaluation of this sensation should include the assessment of medication adherence, stool consistency, and patient-reported symptoms.

### Interpretation of Sex Differences

The observed sex-specific patterns in symptom presentation suggest potential differences in the relative contributions of colonic transit versus evacuation processes.

The higher likelihood of reduced bowel movement frequency in women may be related to slower colonic transit. Age-related changes, including degeneration of colonic smooth muscle and increased collagen deposition, have been reported to reduce high-amplitude propagating contractions, which may prolong colonic transit time [8]. Such mechanisms may contribute to the higher prevalence of reduced bowel movement frequency in older women.

In contrast, the lower likelihood of straining and anorectal obstruction or blockage in women suggests that men may be more likely to report symptoms related to evacuation difficulty. This pattern may reflect differences in symptom generation and reporting rather than differences in physiological sensitivity alone. While women are generally considered to be at higher risk of structural pelvic floor abnormalities, such as rectocele [33], the present findings in a pharmacologically treated population suggest that symptom expression differs, with women more likely to exhibit delayed transit features and men more likely to report outlet-related symptoms.

### Limitations of the Research and Clinical Significance of This Study

The findings of this study should be interpreted with caution in light of several limitations. First, due to the cross-sectional design, temporal relationships between the examined factors and constipation symptoms could not be established; therefore, the observed associations should not be interpreted as causal. Second, this study used a web-based survey with participants recruited from an online research panel, which may introduce selection bias. In addition, the study population was restricted to individuals aged 65 to 74 years who were able to complete an online questionnaire. These factors may limit the representativeness of the sample and reduce the generalizability of the findings to older adults (≥75 y), individuals with cognitive impairment, or those without internet access. Third, key variables—including symptoms, lifestyle factors, treatment goal sharing, medication adherence, and adverse effects—were assessed using self-reported measures. This may introduce measurement error, recall bias, and reporting bias and could result in residual confounding. Moreover, some survey items were developed based on theoretical considerations and expert review, but lack formal reliability and validity data beyond content validation, and were not fully validated against established instruments or objective indicators. In addition, adverse effects were assessed without detailed information on symptom types or severity. Finally, objective physiological indicators, such as colonic transit time and pelvic floor function, were not evaluated, and these unmeasured factors may have influenced the observed associations. Future studies using longitudinal designs, validated multi-item instruments, and objective clinical measures are warranted to confirm the robustness of these findings.

Despite these limitations, the clinical significance of this study lies in the use of self-reported patient data. These findings suggest that the symptoms of chronic constipation may involve multifactorial contributors that cannot be explained by a single mechanism. Rather, multiple factors, including defecatory function, lifestyle habits, and treatment-related processes, contribute differentially to each symptom. In the future, symptom-specific assessments using electronic patient-reported outcomes may provide a basis for more targeted and individualized management strategies in older adults.

### Conclusions

This study demonstrated that in older adults with chronic constipation, less than 3 spontaneous bowel movements per week, straining during bowel movements, a sensation of incomplete evacuation, and a sensation of anorectal obstruction or blockage were each associated with distinct factors. Hard stool is a key factor common to both objective and subjective symptoms, suggesting the importance of focusing on stool consistency as a central element in evaluating constipation.

Additionally, although some associations were not significant after correction for multiple comparisons, symptom-specific associations were observed. These findings suggest that uniform, one-size-fits-all approaches to symptom management may be insufficient in older adults with chronic constipation. Instead, symptom management may benefit from a more symptom-oriented approach that considers underlying pathophysiology, lifestyle factors, and treatment processes.

From a digital health perspective, these results support the use of electronically collected patient-reported outcomes to enable continuous, scalable, and individualized assessment of constipation symptoms. Such approaches may facilitate more targeted and personalized management strategies in real-world clinical settings.

## Supplementary material

10.2196/93531 Multimedia Appendix 1 Questionnaire and supplementary univariable analyses of constipation-related symptoms.

## Abbreviations

AUC: area under the receiver operating characteristic curve
BSFS: Bristol Stool Form Scale
FDR: false discovery rate
OR: odds ratio
